# Fabrication of a Pneumatic Microparticle Concentrator

**DOI:** 10.3390/mi11010040

**Published:** 2019-12-28

**Authors:** Jun Ho Jang, Ok Chan Jeong

**Affiliations:** 1 Institute of Digital Anti-aging, Inje University, Gimhae 50834, Korea; wkdwnsgh0926@naver.com; 2 Department of Biomedical Engineering, Inje University, Gimhae 50834, Korea

**Keywords:** microparticle, pneumatic valve, sieve, concentration, polydimethylsiloxane

## Abstract

We developed a microfluidic platform employing (normally open) pneumatic valves for particle concentration. The device features a three-dimensional network with a curved fluidic channel and three pneumatic valves (a sieve valve (Vs) that concentrates particles and two ON/OFF rubber-seal pneumatic valves that block the working fluid). Double-sided replication employing polydimethylsiloxane (PDMS) was used to fabricate the network, channel, and chamber. Particles were blocked by deformation of the Vs diaphragm, and then accumulated in the curved microfluidic channel. The working fluid was discharged via operation of the two ON/OFF valves. After concentration, particles were released to an outlet port. The Vs pressure required to block solid particles varying in diameter was determined based on the height of the curved microchannel and a finite element method (FEM) simulation of Vs diaphragm displacement. Our method was verified according to the temporal response of the fluid flow rate controlled by the pneumatic valves. Furthermore, all particles with various diameters were successfully blocked, accumulated, and released. The operating pressure, time required for concentration, and concentration ratio were dependent on the particle diameter. The estimated concentration percentage of 24.9 µm diameter polystyrene particles was about 3.82% for 20 min of operation.

## 1. Introduction

Microfluidics and biomedical microelectromechanical systems (BioMEMS) technology [1] have been employed to develop devices that purify and concentrate microparticles used in biological research [2,3,4,5]. Particle traps may be active or passive. Active traps exploit external dielectrophoretic [6,7], magnetophoretic [8,9], acoustic [10,11,12,13], optical [14,15], or thermal [16] forces that act on individual particles, the movement of which is thus precisely controlled. However, the processing rate is low because of the need for physical interaction between the external force and the particles. As the external forces imparted to target particles in microfluidic systems are generally weak, the flow rate must be carefully controlled. Typically, passive microfluidic devices feature micropillars in the microchannels [17,18,19]. Particles are trapped via physical interaction with the streaming working fluid. Such devices are simple to design and can be fabricated inexpensively. However, the micropillars may become clogged as particles accumulate; more complex clog-free devices have thus been developed [20,21,22]. Each device has unique advantages and disadvantages when used to separate microparticles.

Various pneumatically driven microfluidic devices have been developed to trap and release particles using the structural deformation of microstructures [23]. A pneumatically actuated membrane with two different barrier structures was used for handling of the cells [24]. Cells are trapped at the position of the small leakage channels on the first barrier. The leaked fluid with no cells is discharged by a second barrier. When a negative pressure is applied to the membrane, the two barrier structures are lifted, and then the trapped cells are released. A microfiltration mechanism of particles using structural deformation of the thin membrane was developed [25]. The beads or cells are trapped at voids formed in the two corners of a microfluidic channel by the structural difference between the pneumatically inflated thin diaphragm and the microfluidic channel with a rectangular cross section. A pneumatic filtration system was also developed for the size-based separation of particles [26]. It consists of the pneumatic micropump for automatic liquid transport and normally closed valves for the separation of the particles. The filtration mechanism is based on adjustable deformation of the flexible membrane, defining the gap between the microchannel and the floating block structure, which determines the maximum diameter of the bead/cell that can pass through the filter structure. An integrated microfluidic device for dynamic trapping and high-throughput patterning of cells using pneumatic microstructures [27] was investigated. There are two kinds of membrane structures, that is, an array of active U-shaped microstructures for dynamic localization of cells and an umbrella structure for protecting trapped cells in the process of rinsing.

In general, these methods are suitable for handling a limited number of particles when considering the operating methods and structures of the microfluidic platform. The operation conditions such as the flow rate and the magnitude of the compressed air pressure are carefully controlled to prevent unwanted damage to the cells. Further, additional microstructures are also needed to increase cell trap efficiency. A new method is needed to concentrate a large number of particles using a simple and effective method.

Here, we develop a pneumatically driven microfluidic platform for the concentration of massive particles. Our device features a three-dimensional (3D) fluidic network with a curved fluidic channel and chamber structure [28] and three pneumatic valves (a sieve valve (Vs) for particle concentration and two ON/OFF valves that block the working fluid). The 3D fluidic network as a core structure is fabricated via double-sided replication using polydimethylsiloxane (PDMS). The particles of various diameters are concentrated and released using programmed pneumatic operation of the pneumatic valves.

## 2. The Concentrator

### 2.1. Structure

**Figure 1:**
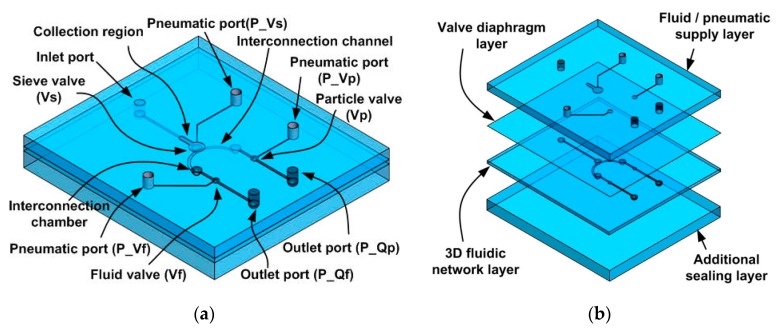
The pneumatically driven microfluidic platform that concentrates particles. (**a**) A schematic of the concentrator. (**b**) Device assembly. (P: port, Q: flowrate, f: fluid, p: particle, V: valve, s: sieve).

Figure 1a shows the device used to concentrate solid particles. The microfluidic platform features three pneumatic valves (a sieve valve (Vs), a fluid valve (Vf), and a particle valve (Vp)) and a 3D fluidic network. The Vs blocks and concentrates particles from fluid. The Vf and Vp discharge only fluid (Qf) during particle concentration and only particles (Qp) after concentration. The fluidic network features a curved fluidic channel and a chamber at the front, as well as an interconnection channel connection with an interconnection chamber that is rectangular in cross section at the back. Both sides are connected via the rectangular and curved chambers. Particles from the inlet port that are blocked by the *V*s accumulate in the collection region of the curved fluidic channel. Particle-free fluid and concentrated particles are discharged via the two outlet ports.

### 2.2. Assembly

Figure 1b shows how the platform is assembled using four PDMS layers: (1) a fluid/pneumatic layer delivering compressed air to the valves and the fluid/particle mixture; (2) a valve diaphragm; (3) three microfluidic channel networks; and (4) a bottom layer for fluidic sealing. Three pneumatic ports supply compressed air to (normally open) pneumatic valves and the inlet/outlet ports in the fluid/pneumatic supply layer. Replica molding was employed to fabricate the two sides of the three microfluidic channel networks.

### 2.3. Operation

**Figure 2:**
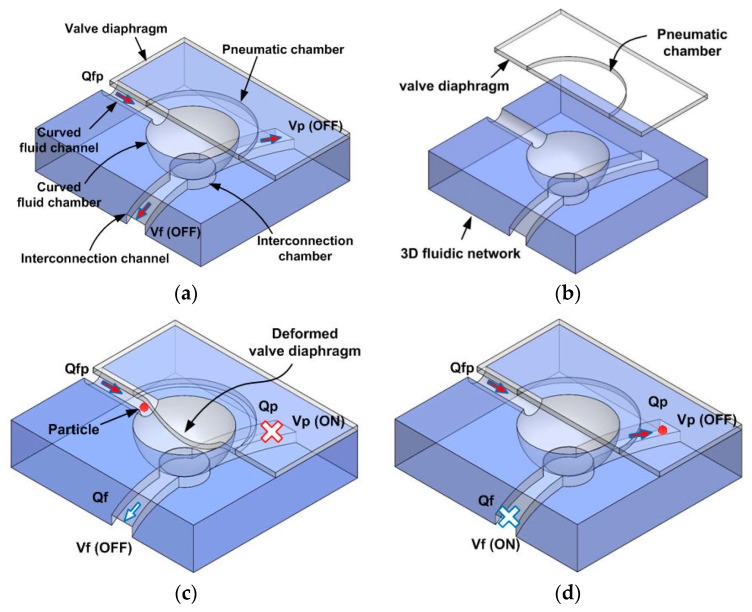
Operation of the particle concentration. (**a**) A schematic of the sieve valve (Vs), (**b**) assembly, (**c**) particle sieving, (**d**) release of concentrated particles.

Figure 2a shows the structure of Vs that blocks particles suspended in the introduced fluid (Qfp). The Vs mainly consists of the valve diaphragm and 3D microfluidic network, as shown in Figure 2b. The curved fluidic channel used for sample introduction (at the front of the 3D fluidic network) is connected at the bottom, via the curved fluid chamber, to the chamber of the rectangular cross section at the back. The fluid flows of particles and pure fluid to the outlet ports, via the interconnection channels, are controlled by the Vp and Vf, respectively. Figure 2c shows the operational method to trap particles. When pressure is applied to the Vs and the Vp, the diaphragms deform and particles in fluid (Qfp) are blocked at the interfacial region between the curved fluid channel and the curved fluid chamber; fluid flow through the interconnection channel (Qp) then ceases. However, unwanted fluid (Qf) is discharged through the open Vf. Figure 2d shows release of the blocked particles. When pressure is applied to the Vf only, the blocked particles (Qp) are discharged through the interconnection channel. Particles of various sizes can be blocked and concentrated by controlling the pressure applied to the Vs.

### 2.4. Fabrication

**Figure 3:**
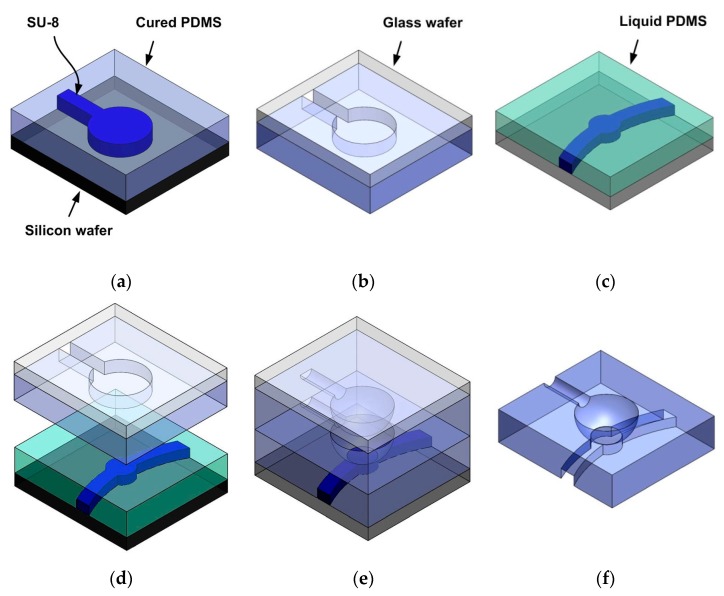
Fabrication of the three-dimensional (3D) fluidic network. (**a**) Replica molding of the curved microfluidic chamber and channel, (**b**) plasma bonding of the cured polydimethylsiloxane (PDMS) layer to a glass wafer, (**c**) pouring of liquid PDMS into an SU-8 mold to form the interconnection channel, (**d**) alignment of the bonded structure and an SU-8 mold, (**e**) replication via thermal curing at 130 °C for 30 min, (**f**) removal of the thermally activated structure and the SU-8 mold.

**Figure 4:**
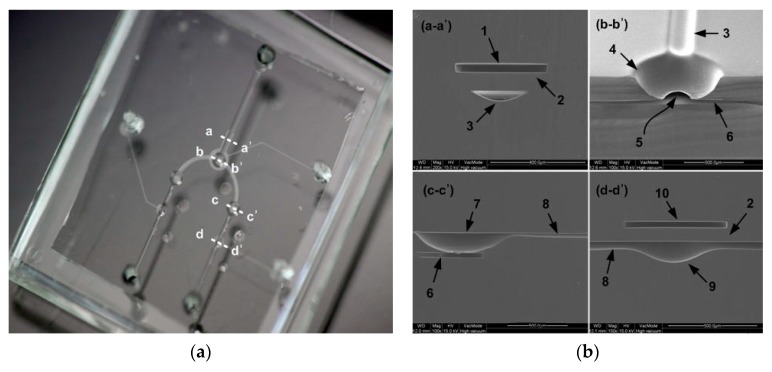
The pneumatically driven microfluidic platform that concentrates particles. (**a**) Fabricated device, (**b**) scanning electron micrographs (SEMs) of device cross sections (**a-a’**: the particle collection region, **b-b’**: Vs, **c-c’**: the interconnection chamber, **d-d’**: the Vp valve, **1**: pneumatic chamber for particle collection, **2**: the valve diaphragm, **3**: the curved particle collection channel, **4**: the curved fluid Vs chamber, **5**: the interconnection port, **6**: the interconnection channel, **7**: the interconnection chamber, **8**: the curved fluid channel to the outlet port, **9**: the fluid chamber for Vp (Vf), **10**: the pneumatic chamber for Vp (Vf)).

Figure 3 shows how the 3D fluidic network (the core structure of the device) is fabricated. Two SU-8 molds are employed for double-sided replication of PDMS layers, forming curved pneumatic valves and microfluidic channels at the front and interconnection channels at the back. First, 10 mL of liquid PDMS was poured into an SU-8 mold, and then thermally activated to form the curved fluid chamber and fluidic channel via spin-coating and curing at 90 °C for 30 min (a) [28]. The cured PDMS layer was peeled off, and the reverse side was bonded to a glass wafer via atmospheric plasma bonding to fabricate a thermally activated mold featuring a sealed air cavity (b). Liquid PDMS (3 mL) was then poured into the SU-8 mold to form the interconnection channel (c). The previously bonded structure was carefully aligned on the liquid PDMS (d). The stacked structure was cured at 130 °C for 30 min (e). During curing of the back structure, a deformable PDMS layer on a thermally activated PDMS mold was inflated by thermal pressure within the air cavity, and the deformed PDMS diaphragm was transferred to the liquid PDMS layer. This allowed fabrication of the curved fluid chamber and the microfluidic channel at the front. After curing, the PDMS mold was separated and the cured microfluidic channel network layer was carefully peeled off the SU-8 mold (f). Figure 4 shows the photograph of the fabricated device and scanning electron micrographs (SEMs) of the cross section. The replica molding employing two SU-8 molds was successful. The dimensions of the microfluidic network are summarized in Table 1.

### 2.5. Finite Element Method (FEM) Simulation

**Figure 5:**
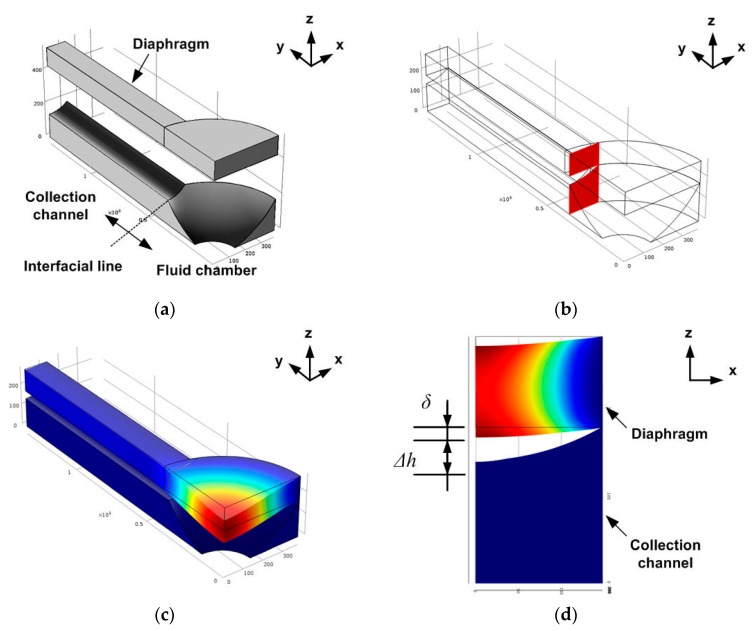
Finite element method (FEM) simulation results. (**a**) The FEM model, (**b**) the cut-plane at the interface of the collection channel and fluid chamber of the Vs, (**c**) structural deformation of the valve diaphragm when a pressure of 10 kPa was applied, (**d**) diaphragm deformation at the cut-plane.

Figure 5 illustrates an FEM simulation of the device performance. The PDMS diaphragm was analyzed using COMSOL Multiphysics (Version 4.2a, COMSOL Inc., Burlington, MA, USA). Figure 5a shows the model used; this included the deformable PDMS diaphragm and fixed structures such as the particle collection channel and fluid chamber of the Vs. Half of the particle collection region and a quarter of the Vs were modeled; structural symmetry was apparent. The dimensions of the FEM model were the same as those of the devices shown in Figure 4 and Table 1. Figure 5b shows the cut-plane (the x–z plane) at the interface of the collection channel and fluid chamber of the Vs. Part of the curved collection channel lay on the surface of the curved fluid chamber; the channel and chamber were replicated from the rectangular and circular diaphragms, respectively. Thus, the cut-plane was placed at the interface between these structures to allow us to explore the valve diaphragm deformation that blocked particles in the collection channel. Figure 5c shows the deflection of the PDMS diaphragm (in the y–z plane) in the particle collection region, and the fluid chamber of the Vs when a pressure of 10 kPa was applied to the Vs chamber. The vertical displacement of the PDMS diaphragm is restricted by the height of the fluid channel used for particle collection and the fluid chamber of the Vs; we thus assumed that the PDMS diaphragm was deformed in the small-strain region. A linear elastic model in solid mechanics featuring a nearly incompressible material was used to simulate deformation of the PDMS diaphragm. The Young’s modulus and Poisson’s ratio of PDMS were set to 664.5 kPa and 0.49, respectively [29]. A free tetrahedral mesh with a maximum size of ~20 μm was used for all domains and had a total of 120,234 elements. A relative tolerance and linearity of the stationary solver was 0.0010 and nonlinear, respectively. Figure 5d shows the deformed PDMS valve at the cut-plane. The deflection of the diaphragm (*δ*) in the cut-plane was maximal at the center of the collection channel. The diameters of particles that might pass through the collection channel were limited by the difference between the diaphragm deflection and the central depth of the collection channel (40 μm). Here, that value is denoted as Δ*h*.

**Figure 6:**
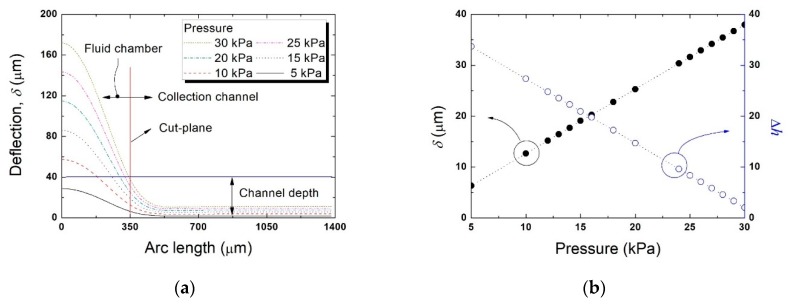
Simulation of polydimethylsiloxane (PDMS) valve diaphragm deflection (in the y–z plane) by applied pressure. (**a**) Deflection of the valve diaphragm within the particle collection region and the fluid chamber of the Vs, (**b**) maximum diaphragm deflection (*δ*) at the cut-plane (x–z plane) and the height difference (Δ*h*) between this deflection and the central depth of the collection channel (40 μm).

Figure 6 summarizes the simulation results. Figure 6a shows the vertical deflection (in the y–z plane) of the valve diaphragm within the particle collection region and the fluid chamber of the Vs. As the pressure applied increased, deflection increased and the fluidic path narrowed. At a pressure of 30 kPa, the interfacial line that blocked particle movement between the collection channel and the sieve fluid chamber was attained. Thus, particles of various sizes could be blocked by adjusting the pressure. Solid particles not deformed by hydraulic pressure could be blocked and thus concentrated. Figure 6b shows the maximum deflection of the diaphragm (*δ*) at the cut-plane (the x–z plane) and the height difference (Δ*h*) between the maximum deflection (δ) and the central depth of the collection channel. The intersection points between the diaphragm deflections (in the y–z plane) and the cut-plane (x–z plane) were obtained from Figure 6a and plotted as maximum diaphragm deflections (*δ* values) in the cut-plane. The *δ* increased as the pressure rose and Δ*h* decreased. Particles were blocked via structural deformation of the valve diaphragm (*δ*) in the cut-plane when the particle diameter was greater than Δ*h*. Thus, as the pressure applied to the Vs increased, the diameters of particles that could pass through the collection channel, to become blocked, were reduced because of increased deflection of the valve diaphragm. Thus, solid particles of various diameters could be blocked and concentrated by tuning deformation of the Vs diaphragm. Linear fitting of Δ*h* (Figure 6b) showed that the minimal pressure *P* required to block particles of diameter *D* was given by (40 – *D*)/1.256.

## 3. Results

### 3.1. Set-Up

We used an accurate pressure controller with four output channels (μFlucon; AMED Co., Seoul, Korea) to control the three pneumatic valves and introduce working fluid into the microfluidic platform. We used the carboxyl polystyrene test particles (Spherotech, Inc., Lake Forest, IL, USA) with three different sizes. The mean diameters of the particles were 24.9 μm, 8.49 μm, and 4.16 μm, respectively. The Vs pressures blocking particles passage was determined based on the simulation results shown in Figure 6b. The pressure applied to the Vp and Vf when blocking the fluidic channel was 18 kPa. Three controller output channels were directly connected to the microvalves. A glass bottle was used to control the flow rate of the working fluid. The bottle was half-filled with water (the working fluid) and a cap with two holes was fitted. Tubes that received compressed air (at 10 kPa) from the controller (one tube) and delivered water to the device input port (the other tube) were then connected to the holes. The working fluid flow rate over time at the outlet port was measured using a liquid flow meter (SLI 1000; Sensirion, Stäfa, Switzerland). Platform operation was observed under an inverted system microscope (IX81; Olympus, Tokyo, Japan).

### 3.2. Flowrate

**Figure 7:**
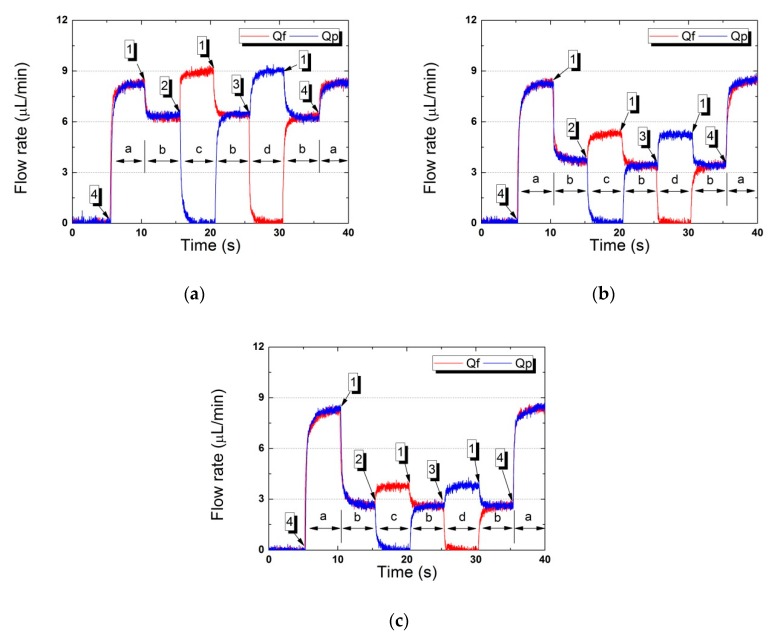
The working fluid flow rate over time as particles became concentrated by the microfluidic platform (a–d show that the positions of the pneumatic valves changed). (**a**) Vs at 15 kPa, (**b**) 26 kPa, (**c**) 30 kPa.

Figure 7 shows the flow rates of the working fluid (water) when three different pressures were applied to Vs. Platform operation (controlled by the three pneumatic valves) was divided into four steps (Table 2). The first step (“state”) was sample loading; fluid was supplied to the platform with all valves open. The flow rates of fluid (Qf) and particles (Qp) were measured at the outlet ports. As the microfluidic channel network exhibited structural symmetry, Qf and Qp were near-identical. When compressed air was delivered to the Vs to block particles (the b state), the flow rates measured at the outlet ports were reduced by hydraulic resistance; the flow path narrowed when the Vs diaphragm became deformed. The flow rates of the working fluid (Qf) and particles (Qp) were very similar; the relative difference between them was less than 2.67%. When concentrating particles (via blocking) with elimination of Qf (the c state), the Vs and Vp were closed, but the Vf was open. The measured flow rate of particle collection (Qp) was near-zero; the microchannel of the particle path was closed by the rubber seal of the pneumatic valve. The flow rate of waste fluid (Qf) was about 1.42-fold that in the b state. When the two parallel channels were in operation, the flow rate doubled. However, other types of hydraulic resistances were in operation (early in the fluidic channel and in the Vs). The overall hydraulic resistance increased because the two parallel fluidic channels became a single channel. Thus, the total flow rate of the working fluid was reduced, but the amount of fluid remained unchanged (Qf).

To collect concentrated particles (d), the pneumatic signals to the valves were reversed. The flow rate of concentrated particles (Qp) was measured only at the outlet port; the flow rate (Qf) became zero because the microchannel was blocked by the pneumatic valve (Vf). The changes in working fluid flow rate over time showed that the microfluidic platform worked well during programmed sequential operation of the pneumatic valves.

### 3.3. Particle Concentration

**Figure 8:**
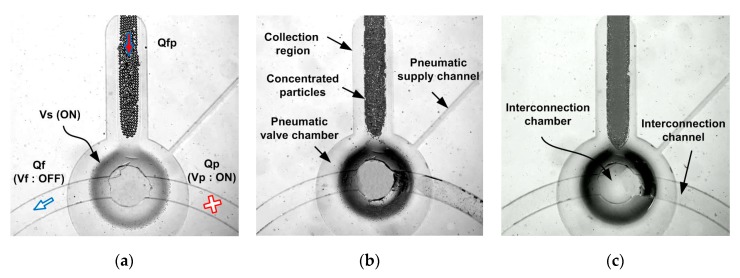
Concentrated particles of various diameters. (**a**) 24.9 μm, (**b**) 8.49 μm, (**c**) 4.16 μm.

Figure 8 shows the captured images of the concentrated particles of various diameters. First, bubble-free deionized water was used to wet the microfluidic path. Then, a particle/fluid mixture was supplied to the inlet port with both the sieve and the Vp under pressure; the particles became concentrated and accumulated in the collection region. Finally, the collected particles were obtained when the Vf (only) was closed. All concentrated particles were released within 4 s. Thus, the device successfully concentrated large numbers of particles. A working flow for the concentration of the 24.9 µm diameter polystyrene particles can be found in SI Movie clip.

### 3.4. Concentration Ratios

Table 3 summarizes the experimental concentration ratios of particles of three different diameters. The concentration ratio is the ratio of the released particle volume (Vol_p) to the sum of the total particle volume (Vol_p) and the waste fluid volume (Vol_f). Particles were released when 160 mL of the waste fluid volume (Vol_f) had exited the outlet port. The Vs pressure increased as particle diameter fell. Also, the released volume of concentrated particles (Vol_p) and the time required for concentration increased as particle diameter reduced. The concentration ratio increased as particle diameter decreased. Particles of various diameters were successfully concentrated. The operation pressure, time required for concentration, and concentration ratio depended on the particle diameter.

### 3.5. Discussion

Herein, a simple and effective method was proposed for purifying and concentrating particles of various sizes. Through the control of pneumatic valves, particles were successfully accumulated and released. The operating pressure, time required for concentration, and concentration ratio were dependent on the particle diameter.

Most active methods focus on the separation of specific target particles from a mixture of purified samples [7,8,9,10,11,12,13]. In contrast to previous studies, the proposed method has some advantages and disadvantages. A smaller number of target particles is used in the dielectrophoretic method [7]. An additional process was required to prepare the particles for enhancing the physical interaction between the external force and the particles [7,9]. The complex design issues of the magnetophoretic separation system should be considered to increase the separation efficiency [7,8]. The separation efficiency of the acoustic method [10,11,12] was greater than that of the other methods. An ultrasonic method achieved a low separation efficiency, although the samples were separated under a high flow rate [13]. Unlike passive methods [17,18,19], no clogging effect was observed [20,21,22] when beads were trapped and accumulated, because there was no passive structure. The proposed method can be used in water pre-treatment for the extraction and concentration of suspended biological particles, as the operation method was not influenced by the physical particle properties.

## 4. Conclusions

We fabricated and tested a particle concentrator with three pneumatic valves. The microfluidic platform had four PDMS layers (a fluid/pressure supply layer, a valve diaphragm layer, a 3D fluidic network layer, and an additional PDMS layer). Three of the layers were fabricated via typical replica molding, but the 3D layer was fabricated via double-sided replication. The curved fluidic channels and chambers in the front were transferred from the deformed diaphragm of a thermally activated PDMS mold. The interconnection channel in the back was replicated via typical soft lithography using PDMS.

The working principle involves particle blocking via deformation of the Vs diaphragm, and control of working fluid flow within the 3D network via sequential operation of the pneumatic valves. When pressure is applied to the Vs, the diaphragm becomes deformed and blocks particles within the curved fluid channel. By controlling the rubber valves, waste fluid and accumulated target particles were released as planned. We evaluated fluid responses under microvalve control over time, and the concentration of particles of various diameters. The required pressures were determined via FEM analysis of Vs diaphragm deformation. The working fluid flow rate was near zero when compressed air was delivered to the ON/OFF valves. The microfluidic channels were successfully closed by the pneumatic rubber valves; the seals were near-perfect. Solid particles were successfully concentrated via programmed valve operation.

## Figures and Tables

**Table 1:** Device dimensions (W: width, D: diameter, H: height).

Device		Width or Diameter (μm)	Height (μm)
1	Pneumatic chamber	400 (D)	38
2	Valve diaphragm	760 (D)	105
3	Curved fluid channel	230 (W)	40
4	Curved fluid chamber for Vs	760 (D)	172
5	Interconnection hole	400 (D)	38
6	Interconnection channel	200 (W)	38
7	Interconnection chamber	760 (D)	172
8	Curved fluid channel	230 (W)	40
9	Fluid chamber for Vp (Vf)	400 (D)	112
10	Pneumatic chamber for Vp (Vf)	600 (D)	38

**Table 2:** Particle concentration by pneumatic valves.

State	Platform Operation	Valve Operation				Flow Rate (µL/min) Depending on Vs Pressures					
						at 15 kPa		at 26 kPa		at 30 kPa	
		Signal	Vs	Vf	Vp	Qf	Qp	Qf	Qp	Qf	Qp
a	Loading	4	OFF	OFF	OFF	8.26	8.31	8.26	8.27	8.22	8.25
b	Blocking	1	ON	OFF	OFF	6.22	6.35	3.66	3.76	2.64	2.65
c	Concentration	2	ON	OFF	ON	8.88	0	5.21	0	3.73	0
d	Release	3	ON	ON	OFF	0	8.98	0	5.23	0	3.76

**Table 3:** Concentration ratios for particles of various diameters.

Particle		Pressure (kPa)			Volume (mL)		Time (min)	Conc. Ratio (%) ^3^
Diameter (μm) ^1^	Number ^2^	Vs	Vf	Vp	Vol_f	Vol_p		Qp/(Qf + Qp) × 100
24.9	934	15	18	18	160	6.6	21	3.96
8.49	23,587	26	18	18	160	7.3	32	4.36
4.16	200,508	30	18	18	160	7.6	47	4.53

^1^ Mean diameter. ^2^ The maximum number of collectible particles. The collection region in the curved fluid channel was modelled as a horizontal cylinder segment and its volume was calculated. The curved channel dimension was used in Table 2. The maximum number of collectible particles was estimated by the volume ratio of the circular segment and a single particle. ^3^ Concentration.
